# OFDM with Index Modulation for Asynchronous mMTC Networks

**DOI:** 10.3390/s18041280

**Published:** 2018-04-21

**Authors:** Seda Doğan, Armed Tusha, Hüseyin Arslan

**Affiliations:** 1 Department of Electrical and Electronics Engineering, Istanbul Medipol University, 34810 Istanbul, Turkey; atusha@st.medipol.edu.tr (A.T.); arslan@usf.edu (H.A.); 2 Department of Electrical Engineering, University of South Florida, Tampa, FL 33620, USA

**Keywords:** asynchronous transmission, massive Machine-Type Communication (mMTC), Orthogonal Frequency Division Multiplexing (OFDM), OFDM with Index Modulation (OFDM-IM)

## Abstract

One of the critical missions for next-generation wireless communication systems is to fulfill the high demand for massive Machine-Type Communications (mMTC). In mMTC systems, a sporadic transmission is performed between machine users and base station (BS). Lack of coordination between the users and BS in time destroys orthogonality between the subcarriers, and causes inter-carrier interference (ICI). Therefore, providing services to asynchronous massive machine users is a major challenge for Orthogonal Frequency Division Multiplexing (OFDM). In this study, OFDM with index modulation (OFDM-IM) is proposed as an eligible solution to alleviate ICI caused by asynchronous transmission in uncoordinated mMTC networks. In OFDM-IM, data transmission is performed not only by modulated subcarriers but also by the indices of active subcarriers. Unlike classical OFDM, fractional subcarrier activation leads to less ICI in OFDM-IM technology. A novel subcarrier mapping scheme (SMS) named as Inner Subcarrier Activation is proposed to further alleviate adjacent user interference in asynchronous OFDM-IM-based systems. ISA reduces inter-user interference since it gives more activation priority to inner subcarriers compared with the existing SMS-s. The superiority of the proposed SMS is shown through both theoretical analysis and computer-based simulations in comparison to existing mapping schemes for asynchronous systems.

## 1. Introduction

Wireless communication systems can be classified into two fundamental categories, namely human-user-based and machine-user-based from the perspective of 5G use cases and applications [1,2]. Current technology gives priority to human-based communications. However, the emerging idea of Massive Machine-Type Communications (mMTC) such as Internet of Things (IoT), vehicle-to-vehicle (V2V), vehicle-to-infrastructure (V2I), control of autonomous vehicles and smart cities with millions of sensors poses various demands for the next-generation networks [3,4,5]. mMTC, where a large number of machine users sporadically communicate with a given base station (BS), leads to asynchronous uplink transmission associated with multi-user interference (MUI). Hence, handling of asynchronous impairments is expected to be one of the most challenging problems for mMTC networks [2,3,6].

Orthogonal frequency division multiplexing (OFDM) has been well studied by academia in the last two decades [7]. It has been shown that OFDM is robust against inter-symbol interference (ISI) with the aid of cyclic prefix (CP), which turns the linear convolution with the channel into a circular convolution [8]. However, OFDM is severely effected from inter-carrier interference (ICI) due to loss of subcarrier orthogonality.

In multi-user OFDM, the users must be aligned in the time and frequency domains in order to maintain the orthogonality between the subcarriers. However, multi-user time alignment is infeasible in asynchronous mMTC-based systems, since signals transmitted from the users at different distances from the BS arrive with different time delays. Time misalignment causes ICI between the users. Furthermore, it is expected that the impact of MUI becomes significant when different power levels are assigned to the machine users, with respect to the applications or used cases [9]. Even if equal power is distributed to the users, as far as signals travel through different paths, power misalignment occurs at the BS.

In literature, 5G candidate waveforms including filter bank multi-carrier (FBMC), generalized frequency-division multiplexing (GFDM) and universal filtered multi-carrier (UFMC) are studied to relax MUI by suppressing out-of-band emission (OOBE) [2,10,11,12]. Moreover, inserting guard-bands between the users is used to further suppress OOBE [9,13]. However, filtering process increases the system complexity and use of guard-bands reduce spectral efficiency. In [14], a new perspective is presented to reduce MUI by clustering of the channel impulse response.

**Figure 1:**
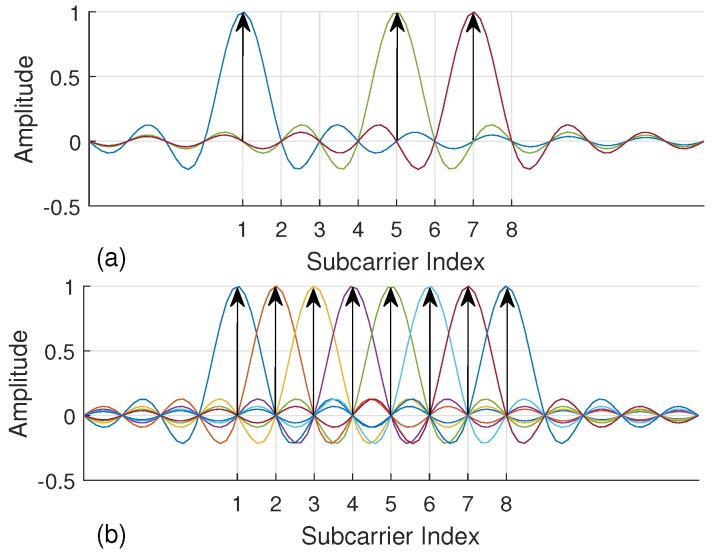
Subcarrier presentation in frequency domain for OFDM and OFDM-IM. Each color refers to a single subcarrier. (**a**) Three out of eight subcarriers are activated in OFDM-IM. (**b**) All of eight subcarriers are utilized in OFDM.

Recently, the proliferation of index modulation (IM) has introduced new research perspectives for 5G wireless systems [15]. At first, IM has been presented as spatial modulation technique (SM) for multiple-input multiple-output (MIMO) systems to convey information by antenna indices [16]. The notion of IM is also extended to OFDM and named as OFDM with index modulation (OFDM-IM), which carries information not only by data symbols but also by the indices of active subcarriers [17,18]. In contrast to conventional OFDM, not all subcarriers are utilized in OFDM-IM. In Figure 1, a simple example is illustrated for an OFDM-IM subblock consisting of eight subcarriers, where three of them are activated to convey data symbols. Extra bits are carried by the indices of active subcarriers to compensate inefficient use of spectrum. In addition, fractional subcarrier activation brings in diversity order as well as less energy consumption [15]. Hence, OFDM-IM provides a flexible and adaptive structure which can be optimized by considering the demands of next-generation communication systems.

Mapping incoming bits to the subcarrier indices is one of the flexible properties of OFDM-IM. In the literature, three subcarrier mapping schemes (SMS) have been proposed to improve error performance and to reduce complexity of the OFDM-IM-based systems. Look-up table (LUT) is the first technique used as a mapping scheme, which uses same storage table at both transmitter and receiver [18]. However, it is not practical for large OFDM-IM subblock sizes. Therefore, Combinatorial method (COM), which does not require storage table, is proposed in [18]. Due to non-uniform subcarrier activation, COM leads to an unequal protection of the transmitted information bits that makes ultimate error performance worse. Hence, equiprobable subcarrier activation (ESA) technique is proposed in [19]. It is observed an enhancement up to 1.9 dB on error rate performance by using ESA for noisy multipath fading channels.

Performance of OFDM-IM is investigated under various impairments by researchers. In [18], it is shown that OFDM-IM under frequency selective fading channels impairment with high mobility is more robust than OFDM. Due to robustness against mobility, it is offered as a candidate for vehicle to X (V2X) communication systems [20]. ICI stemming from carrier frequency offset (CFO) impairment is evaluated by introducing notions of inter-subblock and intra-subblock interference for OFDM-IM [21]. It is observed that OFDM-IM is superior to current technology when the signal is impaired by CFO. In [22], both ICI and ISI is analyzed and mitigated using optimal tone spacing between adjacent subcarriers.

To the best of our knowledge, the performance of OFDM-IM under asynchronous transmission has not been characterized or investigated. In this paper, OFDM-IM is proposed as a candidate solution for uncoordinated mMTC networks. A novel subcarrier mapping scheme (ISA) is proposed to provide further enhancement of OFDM-IM performance for asynchronous systems. It is compared with the current ESA and COM mapping methods. The comparison is performed for various OFDM-IM subblock parameters to evaluate impact of flexibility properties of OFDM-IM. Not only time misalignment but also power difference between the machine users is considered in this study. In addition, ICI analysis is performed and the performance of the OFDM-IM is compared with conventional OFDM in the present of time and power offset between the users.

The remainder of this work is organized as follows. Section 2 introduces the multi-user OFDM-IM system model for asynchronous transmission. In Section 3, ICI analysis is provided for OFDM-IM. In Section 4, existing SMS are revisited and a novel mapping technique is proposed. Numerical results are given in Section 5. Finally, some concluding remarks are provided for OFDM-IM technology with mMTC in Section 6.

## 2. System Model

**Figure 2:**
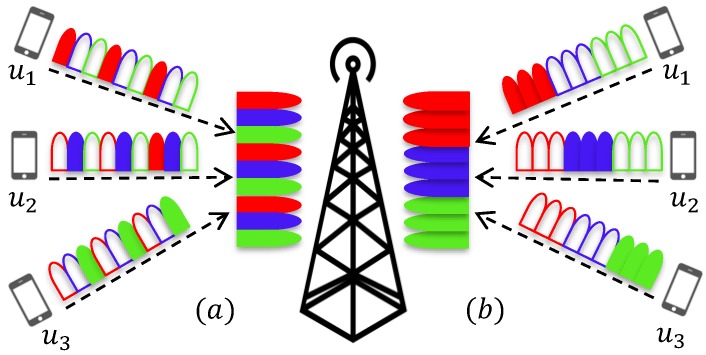
Uplink system model and user-subblock assignment methods: (**a**) interleaved; (**b**) localized. In frequency domain, each user’s band is shown with a different color. For a given user, filled bands represent used OFDM-IM subblock, which contains more than one subcarrier.

This section introduces an uplink system model where *U* users independently communicate with the base station (BS) through a frequency selective channel. A simple uplink system example is presented in Figure 2. Each user’s information is modulated with OFDM-IM. A total of *N* subcarriers is equally split between the users, and $N_{u}=\frac{N}{U}$ subcarriers are dedicated to *u*-th user, with 1≤u≤U. Assignment of OFDM-IM subblocks to the users can be performed in two ways, either interleaved-based or localized-based. Interleaved-based assignment mixes the users’ subblocks, while localized-based assignment successively places each user’s subblocks, as visualized in Figure 2.

**Figure 3:**
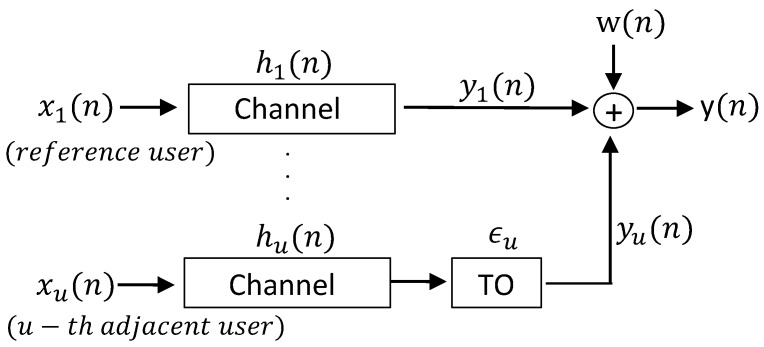
Baseband equivalent model of the uplink system by considering time offset between the users.

In Figure 3, time domain signal that belongs to *u*-th user is expressed as $x_{u}\left(n\right)$. It is assumed that $x_{1}\left(n\right)$, which reaches first to the BS, is considered to be reference signal for the BS. Each user’s signal arrives to the BS with a different time offset (TO) $\epsilon_{u}$ with respect to $x_{1}\left(n\right)$ since they can be placed at different distances from the BS or can be transmitted at different times. The transmitted signal from each user passes through its own channel $h_{u}\left(n\right)$. All the channels are uncorrelated with each other. Later, individual signals $y_{u}\left(n\right)$ transmitted from all the users is superimposed, and additive white Gaussian noise (AWGN) w(n) is added to the superimposed signal y(u). Due to the time misalignments, orthogonality between the machine users cannot be maintained anymore. Therefore, ICI between the users occurs and degrades the system performance. Table 1 presents symbols used in the study and their descriptions. Further insights about asynchronous mMTC transmission with OFDM-IM are given in following subsection.

### OFDM-IM Transmission Model

In this work, it is considered *N* size OFDM-IM block, where subcarriers are equally split into *G* subblocks. Each subblock consists of $s=\frac{N}{G}$ subcarriers and *v* out of *s* are selected to transmit *M*-ary data symbols with 1≤v<s. As mentioned in Section 1, in contrast with conventional OFDM (v=s), not all subcarriers are utilized for *M*-ary symbols. Hence, the loss of spectral efficiency is compensated by the used subcarrier indices that convey additional information bits.

**Figure 4:**
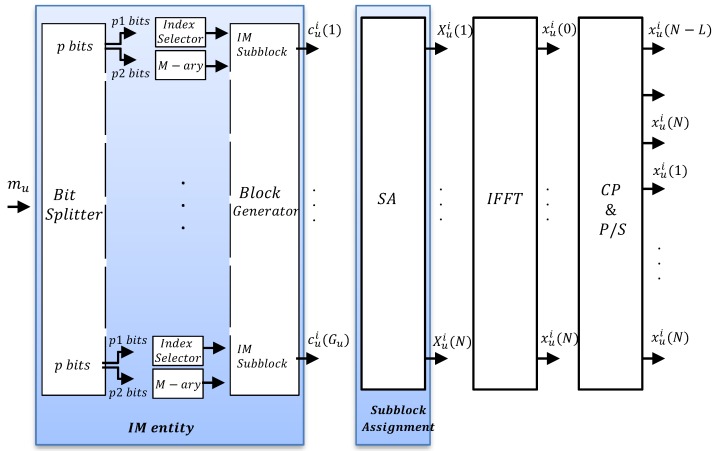
Block diagram of asynchronous OFDM-IM transmitter for *i*-th block of *u*-th user.

In multi-user transmission, each user has a total of $G_{u}=\frac{N_{u}}{s}$ available subblocks to carry $m_{u}$ bit stream, with $1\leq G_{u}\leq G$. When all the subcarriers are assigned to one user, $G_{u}$ equals to *G*. Block diagram for asynchronous OFDM-IM transmitter is shown in Figure 4. Each OFDM-IM subblock consists of $p=\frac{m_{u}}{G_{u}}$ bit stream, which is divided into $p_{1}$ and $p_{2}$ bits. The indices of active subcarriers are defined from $p_{1}$ bit stream, while remaining $p_{2}$ bit stream is mapped to conventional *M*-ary symbols $\left\{d_{1},\cdots ,d_{v}\right\}\in M_{ary}$, which are carried by the activated subcarriers. Division of the *p* bit stream is illustrated by “IM” entity in Figure 4. The indices of active subcarriers of *u*-th user for *l*-th subblock are defined as

(1) $$\xi_{u}^{l}=[j_{u}\left(l,1\right),j_{u}\left(l,2\right),\cdots ,j_{u}\left(l,v\right){]}_{1\times v}$$

where $j_{u}\left(l,v\right)\in \left[1,2,\cdots ,s\right]$ for $l=1,\cdots ,G_{u}$. Thus, total number of conveyed bits per OFDM-IM subblock is calculated as

(2) $$p=p_{1}+p_{2}=\left\lfloor log_{2}\left(C\left(s,v\right)\right)\right\rfloor +vlog_{2}\left(M\right)$$

where ⌊.⌋ and C(s,v) denote floor function and binomial coefficient, respectively. The number of transmitted bits per user is

(3) $$m_{u}=G_{u}p=G_{u}\left\lfloor log_{2}\left(C\left(s,v\right)\right)\right\rfloor +G_{u}vlog_{2}\left(M\right).$$

*l*-th subblock $c_{u}^{i}\left(l\right)$ belongs to *i*-th data block of *u*-th user is represented as

(4) $$c_{u}^{i}\left(l\right)=[c_{u}^{i}\left(l,1\right),c_{u}^{i}\left(l,2\right)\cdots ,c_{u}^{i}\left(l,s\right){]}_{1\times s}$$

where $c_{u}^{i}\left(l,s\right)\in \left\{0,M_{ary}\right\}$. $M_{ary}$ represents the data symbols. Later, as illustrated by “Block Generator” in Figure 4, $G_{u}$ subblocks are combined to form *i*-th data block of *u*-th user $c_{u}^{i}$ expressed as follow

(5) $$c_{u}^{i}=[c_{u}^{i}\left(1\right),\cdots ,c_{u}^{i}\left(l\right),\cdots ,c_{u}^{i}\left(G_{u}\right){]}_{1\times N_{u}}.$$

$V=vG_{u}$ out of $N_{u}=sG_{u}$ subcarriers carry M-ary symbols and the rest equal to zero. “IM” entity in Figure 4 full demonstrates the process of generating the frequency domain data samples for *u*-th user.

Once $c_{u}^{i}$ is generated, it passes through the multi-user “Subblock assignment” (SA) entity, as in Figure 4. SA performs either localized assignment or interleaved assignment for $c_{u}^{i}$, and inserts *N*-$N_{u}$ zeros to the subcarriers assigned to the other users. Then, *i*-th OFDM-IM block of *u*-th user is generated as follow

(6) $$X_{u}^{i}=[0,\cdots ,c_{u}^{i}\left(1\right),\cdots ,0,c_{u}^{i}\left(l\right),\cdots ,0,c_{u}^{i}\left(G_{u}\right),\cdots ,0{]}_{1\times N}.$$

Time domain samples for *i*-th block of *u*-th user are obtained by inverse-Fast Fourier Transform (IFFT) process shown in Figure 4 as

(7) $$x_{u}^{i}\left(n\right)=\sum_{k=0}^{N-1}X_{u}^{i}\left(k\right)e^{j2\pi nk/N},0\leq n\leq N-1.$$

A cyclic prefix (CP) with length L is appended to the beginning of $x_{u}^{i}\left(n\right)$ to prevent inter-symbol interference (ISI) due to time dispersion of the channel [8]. Time domain signal of *u*-th user $x_{u}\left(n\right)$ passes through multipath channel. The signal experiences Rayleigh fading. Channel impulse response coefficients between *u*-th user and the BS for *i*-th block are characterized as

(8) $$h_{u}^{i}\left(n\right)=\sum_{r=0}^{L_{tap}-1}g_{u}^{i}\left(\tau_{r}\right)\delta (n-\tau_{r})$$

where $L_{tap}$ denotes total number of taps, *r* is the path index and $\tau_{r}$ is the delay of the *r*-th path. It is assumed that maximum excess delay of the channel is smaller than CP size, and path gains $g_{u}^{i}$ are Gaussian random variables with distribution $CN(0,1/L_{tap})$. The signal $x_{u}\left(n\right)$ is received as

(9) $$y_{u}\left(n\right)=x_{u}\left(n\right)*h_{u}\left(n\right)$$

where ∗ denotes convolution process. At the BS, signal transmitted from all the machine users are superimposed as follow

(10) $$y\left(n\right)=\sum_{u=0}^{U-1}y_{u}\left(n-\epsilon_{u}\right)+w\left(n\right).$$

w(n) is AWGN with distribution of $CN(0,N_{o}/2)$.

At the receiver, time offset $\epsilon_{u}$ is removed from the superimposed signal to obtain the signal belonging to *u*-th user. Fast Fourier Transform (FFT) is applied to obtain the frequency domain samples Y(k). Then, deassignment process, which refers to the inverse process of the SA, is applied to get only *u*-th user data blocks $c_{u}$. The indices of active subcarriers are detected by using maximum likelihood (ML) or log-likelihood ratio (LLR) detectors. ML detector checks all the possible subcarriers combinations and information symbols to find the most optimum joint decision. LLR receiver first detects active subcarriers and then information symbols carried by the detected subcarriers are demodulated [18].

## 3. ICI Analysis in OFDM-IM Systems

**Figure 5:**
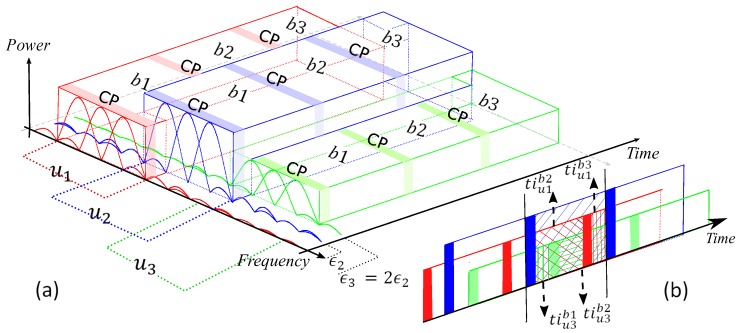
(**a**) Time, frequency and power domain illustration of three users’ signals. Each user’s signal is shown by different color. (**b**) Time domain representation of the superimposed signal at the BS.

Consider a system model which includes U=3 users with 3 OFDM-IM blocks to analyze ICI because of time offset ϵ between the users. These users transmit sporadically in adjacent bands with different transmit power levels, as illustrated in Figure 5a. For the sake of simplicity, it is assumed that equal time offset between the adjacent users. Notations of b1, b2 and b3 in the figure denote first, second and third OFDM-IM block, respectively.

In [6], ICI model is calculated for OFDM systems under time misalignment. Besides time offset, the model is modified for uncoordinated OFDM-IM systems by considering the fact that power difference between the machine users. In contrast to OFDM, only the active subcarriers of the users’ cause ICI in OFDM-IM. Therefore, the indices of interferer subcarriers belong to ξ.

In our calculations, ${ti}_{u_{y}}^{u_{x}}$ shows the interference coming from $u_{x}$-th user to the $u_{y}$-th user while ${ti}_{u_{x}}^{bl}$ denotes the interference caused by *l*-th block of $u_{x}$-th user. $\xi_{u}$ denotes the active subcarrier indices of *u*-th user for all the subblocks. Figure 5b shows the superimposed signal at the BS. As illustrated in the figure, time domain interference for the 2-nd symbol of 2-nd user ${ti}_{u_{2}}$ is calculated as

(11) $$ti_{u_{2}}=ti_{u_{2}}^{u_{1}}+ti_{u_{2}}^{u_{3}},$$

where $ti_{u_{2}}^{u_{1}}=ti_{u_{1}}^{b2}+ti_{u_{1}}^{b3}$ and $ti_{u_{2}}^{u_{3}}=ti_{u_{3}}^{b1}+ti_{u_{3}}^{b2}$, and they are expressed as

(12) $$ti_{u_{2}}^{u_{1}}\left(n\right)=\sum_{n=0,n\in \xi_{1}}^{\epsilon -1}\sqrt{\Delta p_{12}}\left(-x_{1}^{b2}\left(n\right)+x_{1}^{b3}\left(n\right)\right),$$

(13) $$ti_{u_{2}}^{u_{3}}\left(n\right)=\sum_{n=N-\epsilon +L,n\in \xi_{3}}^{N-1}\sqrt{\Delta p_{32}}\left(-x_{3}^{b2}\left(n\right)+x_{3}^{b1}\left(n\right)\right).$$

where $\Delta p_{12}$ and $\Delta p_{32}$ refers to power difference between the 1-st and 2-nd user, and the 3-rd and 2-nd user, respectively.

In the frequency domain, ICI $I_{2}$ is calculated by taking FFT for $ti_{u_{2}}$, and it is expressed as

(14) $$\begin{array}{c} I_{2}\left[k\right]=\sum_{k=0}^{N_{u}-1}ti_{u_{2}}^{u_{1}}\left(n\right)e^{-j2\pi kn/N}+\sum_{k=2N_{u}}^{3N_{u}-1}ti_{u_{2}}^{u_{3}}\left(n\right)e^{-j2\pi kn/N} \end{array}$$

ICI for first user $I_{1}$ and third user $I_{3}$ is obtained as

(15) $$\begin{array}{c} I_{1}\left[k\right]=\sum_{k=N_{u}}^{2N_{u}-1}ti_{u_{1}}^{u_{2}}\left(n\right)e^{-j2\pi kn/N}+\sum_{k=2N_{u}}^{3N_{u}-1}ti_{u_{1}}^{u_{3}}\left(n\right)e^{-j2\pi kn/N}, \end{array}$$

(16) $$\begin{array}{c} I_{3}\left[k\right]=\sum_{k=0}^{N_{u}-1}ti_{u_{3}}^{u_{1}}\left(n\right)e^{-j2\pi kn/N}+\sum_{k=N_{u}}^{2N_{u}-1}ti_{u_{3}}^{u_{2}}\left(n\right)e^{-j2\pi kn/N}. \end{array}$$

As seen in the Figure 5b, by considering both time and power offset $ti_{u_{1}}^{u_{2}},ti_{u_{1}}^{u_{3}},ti_{u_{3}}^{u_{1}}$ and $ti_{u_{3}}^{u_{2}}$ can be easily extracted as

(17) $$ti_{u_{1}}^{u_{2}}\left(n\right)=\sum_{n=N-\epsilon +L,n\in \xi_{2}}^{N-1}\sqrt{\Delta p_{21}}\left(-x_{2}^{b2}\left(n\right)+x_{2}^{b1}\left(n\right)\right)$$

(18) $$ti_{u_{1}}^{u_{3}}\left(n\right)=\sum_{n=N-2\epsilon +L,n\in \xi_{3}}^{N-1}\sqrt{\Delta p_{31}}\left(-x_{3}^{b2}\left(n\right)+x_{3}^{b1}\left(n\right)\right)$$

(19) $$ti_{u_{3}}^{u_{1}}\left(n\right)=\sum_{n=0,n\in \xi_{1}}^{2\epsilon -1}\sqrt{\Delta p_{13}}\left(-x_{1}^{b2}\left(n\right)+x_{1}^{b3}\left(n\right)\right)$$

(20) $$ti_{u_{3}}^{u_{2}}\left(n\right)=\sum_{n=0,n\in \xi_{2}}^{\epsilon -1}\sqrt{\Delta p_{23}}\left(-x_{2}^{b2}\left(n\right)+x_{2}^{b3}\left(n\right)\right)$$

In this paper, it is considered that subcarriers belong to *u*-th user are orthogonal to each other while machine users’ subcarriers are interfering with each other due to the time offset between them. For this reason, interference coming from other users to *u*-th user is mainly determined by its adjacent users’ edge subcarriers. Since the subcarriers are sinc functions in frequency domain, inner subcarriers’ sidelobes have less impact on the ICI compared to the edge subcarriers, as explained in [13,23,24]. Therefore, more users can be considered, but the interference coming from users that are not adjacent with the *u*-th user becomes much smaller.

## 4. OFDM-IM Subcarrier Mapping Schemes

In this section, the existing SMS-s in the literature for OFDM-IM are revised, and the proposed mapping scheme ISA is explained in details.

### 4.1. Existing SMS-s

#### 4.1.1. LUT

The method requires at both transmitter and receiver side a look-up table with the size $d=2^{p_{1}}$ to store all possible combinations of the active subcarrier indicies ξ with respect to $p_{1}$-bit stream. An example of LUT with $p_{1}=2$, v=2 and s=4 is illustrated in Table 2. β(z) denotes bit streams corresponding to each index combination ξ(z), with $z\in [1,2\;...\;2^{p_{1}}]$. The size of look-up table significantly increases the system complexity with the increase of the $p_{1}$. Therefore, LUT scheme become infeasible for large $p_{1}$.

At the receiver, ML detector is used to make a joint decision for active subcarrier indices with *M*-ary data symbols [18].

#### 4.1.2. COM

In contrast to LUT method, storage tables at the transmitter and receiver are not required. It assigns a specific lexicographically ordered sequence $J\left(z\right)=\left\{\alpha_{v},\;...,\;\alpha_{1}\right\}$ with α∈{0,...,s−1} for each possible index combination ξ(z). The β(z)-bit stream is converted to a natural number *E*, which is converted to a specific J(z) sequence as follow

(21) $$E=C\left(\alpha_{v},v\right)\;+\;...\;+C\;\left(\alpha_{1},1\right),\;\;\;\;s>\alpha_{v}>...\;\alpha_{1}\geq 0.$$

To select α components, we start from the condition that satisfies $E\geq C(\alpha_{v},v)$ and then choose the maximal $\alpha_{v-1}$ that satisfies $E-C\left(\alpha_{v},v\right)\geq C\left(\alpha_{v-1},v-1\right)$ until v=1 and then the index combination is obtained as ξ(z)=J(z)+1. Detailed information about COM can be found in [18].

In the receiver, firstly ξ(z) is identified for a given subblock by using LLR detector, and J(z)=ξ(z)−1 is mapped to its corresponding decimal number *E*, which passes through bit to decimal converter to get β(z) bit stream.

**Figure 6:**
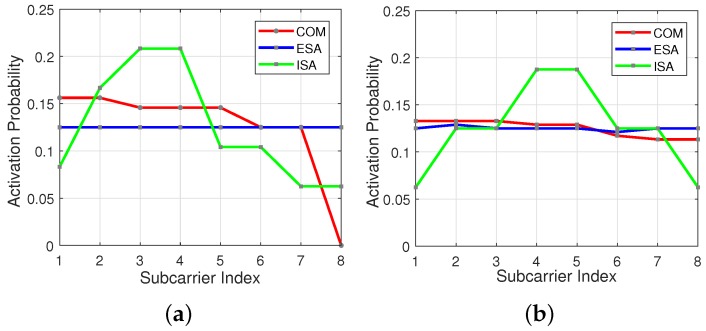
Subcarrier usage probability within an OFDM-IM subblock for the three SMS-s regarding to different (s,v). (**a**) s=8 and v=3. (**b**) s=8 and v=4.

In Figure 6a,b subcarrier activation probability is represented by the red line for COM scheme. As seen in the figures, initial subcarriers have higher usage probability in comparison to the last ones, especially for s=8 and v=3.

#### 4.1.3. ESA

In contrast with COM method, ESA offers as much as possible equiprobable subcarrier activation opportunity as illustrated in Figure 6a,b by the blue line [19]. A small table named as adjacent subcarrier distance vector (ASDV) is present at both transmitter and receiver to find C(s−1,v−1) basic combinations $\xi_{b}$, which belongs to ξ. By using column cyclic shift s−1 new active subcarrier combinations are generated from the $\xi_{b}$. The new combinations have the same ASDV with the corresponding basic pattern $\xi_{b}$. Note that some index combinations generated from cyclic shift of $\xi_{b}$-s can be the same. In this case, ASDV considers only one from repeated patterns and disregards the rest. This idea successively is applied all over $\xi_{b}$ until we get all $2^{p_{1}}$ possible subcarrier combinations ξ. Selection of the basic patterns $\xi_{b}$ are explained in [19]. At the receiver side, LLR receiver is used to find ξ(z) that is mapped to β(z)-bit stream for a given subblock.

### 4.2. Proposed SMS: ISA

Aforementioned SMS-s are designed for synchronous communication systems, which leads to equal noise power level at each subcarrier. Therefore, in this study new mapping scheme ISA, which stands for inner subcarrier activation, is proposed and explained to alleviate the ICI due to sporadic transmission in mMTC.

ISA scheme gives a higher activation probability to the subcarriers located at the center part of the OFDM-IM subblock, as illustrated in Figure 6a,b by the green line. OOBE coming from inner subcarrier is less than that of edge subcarriers [23]. Therefore, each user experiences less interference from its adjacent users.

ISA scheme is based on the COM scheme, which directly maps β(z) bits to subcarrier indices ξ(z), and vise versa. As calculated in line 1 of Algorithm 1, a subblock with *s* subcarriers is divided into two parts, where first part and second part contains $s_{1}$ subcarriers and $s_{2}$ subcarriers, respectively. $v_{1}$ subcarriers and $v_{2}$ subcarriers are activated to carry data information symbols. Indices for $v_{1}$ active subcarriers $\xi_{1}\left(z\right)$ are selected by flipped version of COM method, which is calculated from line 4 through 6. Conventional COM method is used to select $v_{2}$ subcarrier indices $\xi_{2}\left(z\right)$ as shown in line 7. Consequently, indices of active subcarriers for β(z) are composed of $\xi_{1}\left(z\right)$ and $\xi_{2}\left(z\right)$, as shown in line 8. In ISA, $p_{1}$ equals $\left\lfloor log_{2}\left(C\left(s_{1},v_{1}\right)\right)\right\rfloor +\left\lfloor log_{2}\left(C\left(s_{2},v_{2}\right)\right)\right\rfloor \leq \left\lfloor log_{2}\left(C\left(s,v\right)\right)\right\rfloor$. This results in less spectral efficiency for some combinations of *s* and *v*.

**Algorithm 1** ISA mapper.
1:$s_{1}=\left\lfloor s/2\right\rfloor$, $s_{2}=s-s_{1}$       ▹ # of subcarriers for each part2:$v_{1}=\left\lfloor v/2\right\rfloor$, $v_{2}=v-v_{1}$    ▹ # of active subcarriers for each part3:$\beta \left(z\right)=\left[\beta_{1}\left(z\right)\;\beta_{2}\left(z\right)\right]$            ▹ Incoming bit stream4:$c=\left[s_{1}-1:-1:0\right]$   5:$\xi_{1}\left(z\right)=COM\left(\beta_{1}\left(z\right),s_{1},v_{1}\right)$   6:$\xi_{1}\left(z\right)=1+c\left(\xi_{1}\left(z\right)\right)$          ▹ Flipped version of COM7:$\xi_{2}\left(z\right)=v_{1}+COM\left(\beta_{2}\left(z\right),s_{2},v_{2}\right)$            ▹ COM8:$\xi \left(z\right)=\left[\xi_{1}\left(z\right)\;\xi_{2}\left(z\right)\right]$         ▹ Activated subcarrier indices


At the receiver, LLR detectors are used to know the active subcarrier indices ξ(z), as shown in line 3 through 6 of Algorithm 2.

**Algorithm 2** ISA demapper.
1:$s_{1}=\left\lfloor s/2\right\rfloor$, $s_{2}=s-s_{1}$               ▹ # of subcarriers for each part2:$v_{1}=\left\lfloor v/2\right\rfloor$, $v_{2}=v-v_{1}$            ▹ # of active subcarriers for each part3:$c^{{}^{\prime }}=\left[0:1:s_{1}-1\right]$   4:$\xi_{1}\left(z\right)=LLR\left(s_{1},v_{1}\right)$   5:$\xi_{1}\left(z\right)=\xi_{1}\left(z\right)-1,\xi_{1}\left(z\right)=sort\left(\xi_{1}\left(z\right){,}^{\prime }ascend^{\prime }\right)$   6:$\xi_{1}\left(z\right)=c^{{}^{\prime }}\left(\xi_{11}\right)$        ▹ Detecting active subcarrier indices for first s1 subcariers7:$\xi_{2}\left(z\right)=LLR\left(s_{2},v_{2}\right)$    ▹ Detecting active subcarrier indices for last s2 subcariers8:
$J_{z1}=\xi_{1}\left(z\right)-1\to E_{1}\to \beta_{1}\left(z\right)\;$
9:
$J_{z2}=\xi_{2}\left(z\right)-1\to E_{2}\to \beta_{2}\left(z\right)\;$
10:$\beta \left(z\right)=\left[\beta_{1}\left(z\right)\;\beta_{2}\left(z\right)\right]$                       ▹ Bit stream


The receiver first calculates the LLR values with respect to each subcarrier as

(22) $$LLR\left(k\right)=log\frac{P(A_{k})}{P(\bar{A_{k}})}+\frac{{|Y\left(k\right)}^{2}|}{N_{0}^{\prime }}+log\frac{1}{M}\sum_{m=1}^{M}\frac{|Y\left(k\right)-H_{u}\left(k\right)d_{m}|}{N_{0}^{\prime }},$$

where $P(A_{k})$ and $P(\bar{A_{k}})$ denotes the probability of *k*-th subcarrier being active and inactive, respectively. $N_{0}^{\prime }=I_{u}+N_{0}$ shows total distortion of the system due to both ICI and noise, and $H_{u}\left(k\right)$ is channel frequency response (CFR) for *u*-th user. After calculation of LLR values for a subblock, *v* out of them with highest LLR define the active subcarriers. The subcarrier index patterns are converted to lexicographically ordered sequences $J_{z1}$ and $J_{z2}$. By using Equation (21), these sequences are mapped to decimal numbers $E_{1}$ and $E_{2}$. Then, $E_{1}$ and $E_{2}$ undergo decimal-to-bit converter to obtain $\beta_{1}\left(z\right)$ and $\beta_{2}\left(z\right)$ bit streams as illustrated in line 7 and 8, respectively. β(z) bit stream is a concatenation of $\beta_{1}\left(z\right)$ and $\beta_{2}\left(z\right)$, as shown in line 9.

Due to the fact that proposed ISA mapper is based on COM mapper, ISA does not bring additional complexity to the system. Unlike LUT and ESA schemes, storage tables are not required for ISA. Moreover, ISA technique gives higher activation probability $P(A_{k})$ to the inner subcarriers with low $N_{0}^{\prime }$ and vice versa. Therefore, the reliability of calculated LLR values is maximum for asynchronous transmission with the aid of ISA regarding to Equation (22). In other words, detection performance of the active subcarriers under asynchronous transmission is increased by ISA.

## 5. Numerical Results and Discussion

This section is dedicated to evaluating the performance of OFDM-IM and OFDM-based systems for asynchronous mMTC networks. Theoretical results for ICI due to both time offset and power difference between the users are first validated by computer-based simulations. Secondly, BER performance for OFDM-IM with three different subcarrier mapping schemes including COM, ESA and ISA are shown to compare their performance for uncoordinated networks. In this study, we assume three users are sporadically transmitting to the BS. Available *N* = 120 subcarriers are equally split between the users. The system is tested over $L_{tap}$ = 10 tap frequency-selective Rayleigh fading channel. A CP size is adjusted as L=30 to prevent ISI for each user. BPSK modulation is used for the machine users. MATLAB software is used for the simulations.

In all simulations, two different subblock parameters are preferred to make a proper comparison between the subcarrier mapping methods. OFDM-IM with ESA for subblock parameters *s* = 8 and *v* = 3 offers the best performance in comparison to COM, since it benefits the most from frequency selectivity of the channel. On the other hand, the performance of ESA becomes similar to COM for the parameters *s* = 8 and *v* = 4 due to loss of selectivity, which is caused by usage of almost all subcarrier combinations [19]. In addition, two different time offset between the users are considered. Minimum and maximum time offset ϵ are adjusted as 24 and (N+L)/2=75, respectively. Power differences between the users obey uniform distribution in a range of 2 dB and 7 dB.

**Figure 7:**
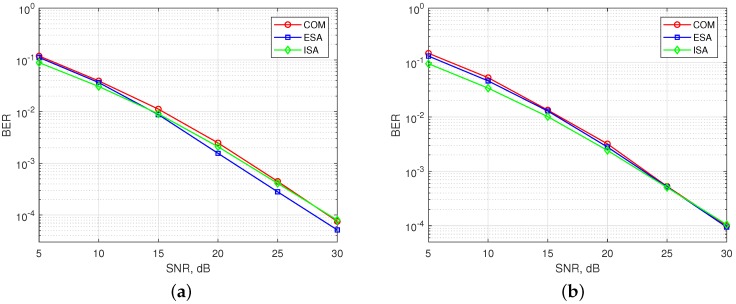
BER performance of synchronous multi-user OFDM-IM regarding to the three SMS-s. (**a**) SMS-s with s=8 and v=3. (**b**) SMS-s with s=8 and v=4.

In Figure 7, BER performance of existing SMS-s and the ISA are simulated for synchronous communication, where all users arrive to the BS at the same time (ϵ=0). Figure 7a shows the results for OFDM-IM with (s=8,v=3). ESA mapper is superior to COM mapper as aforementioned. BER performance of ISA lies in between COM with ($s_{1}$, $v_{1}$) and COM with (*s*, *v*) because of subblock division property. Therefore, the performance of ISA is the best for low signal-to-noise ratio (SNR). Its performance goes near to COM as SNR increases. In Figure 7b, obtained results are illustrated for (s=8,v=4). The performance of ESA is similar to COM [19].The performance of ISA is almost the same with ESA and COM for high SNR, while it outperforms for low SNR due to subblock division.

**Figure 8:**
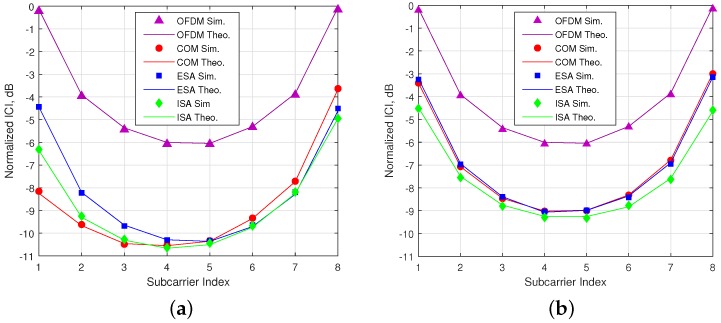
ICI analysis for OFDM and OFDM-IM regarding to three SMS-s. (**a**) SMS-s with s=8 and v=3. (**b**) SMS-s with s=8 and v=4.

In Figure 8, it is shown that theoretical calculations of ICI for both OFDM-IM and conventional OFDM perfectly match with computer-based simulations. The simulation results are obtained under maximum time offset ϵ=Max for 2-nd user, who has less power in comparison to others. As seen in the figure, OFDM-IM is exposed to less ICI thanks to partial subcarrier activation under asynchronous transmission. In the Figure 8a, the most exposed to ICI is ESA, since it has higher probability of edge subcarrier usage as shown in Figure 6a. COM experience minimum ICI for initial subcarriers due to lower usage probability of last subcarriers of the previous user. On the other hand, last subcarriers of subblock are exposed to maximum ICI due to higher usage probability of initial subcarriers of the following user. Proposed method ISA encounters less ICI because of its lower usage probability for edge subcarriers.The obtained ICI results are inversely proportional to the edge subcarrier usage probability within the subblock. According to activation probability for SMS-s with (s=8, v=4) as in the Figure 6b, ISA faces minimum ICI, as illustrated in Figure 8b.

**Figure 9:**
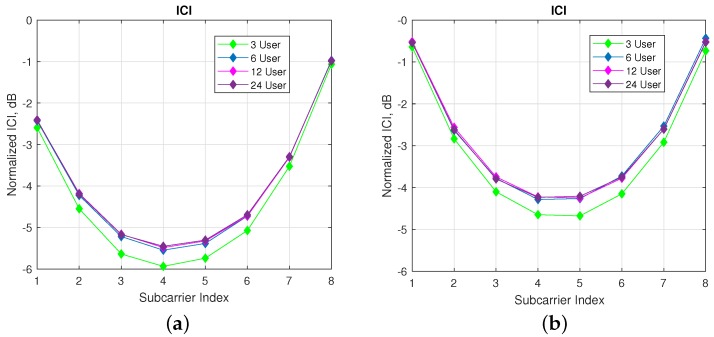
ICI analysis for ISA SMS regarding to various number of machine users. (**a**) ISA SMS with s=8 and v=3. (**b**) ISA SMS with s=8 and v=4.

In Figure 9, ICI analyzes on reference user are performed regarding different number of machine users with a fixed number of subcarriers per user. As seen in the Figure 9a,b, nearly 0.5 dB more interference is observed for 6 users compared with 3 users case. For more than 6 users, there is a very slit increase in the ICI. Hence, the amount of the interference coming from far users proportionally decays with the increase in the number of users.

**Figure 10:**
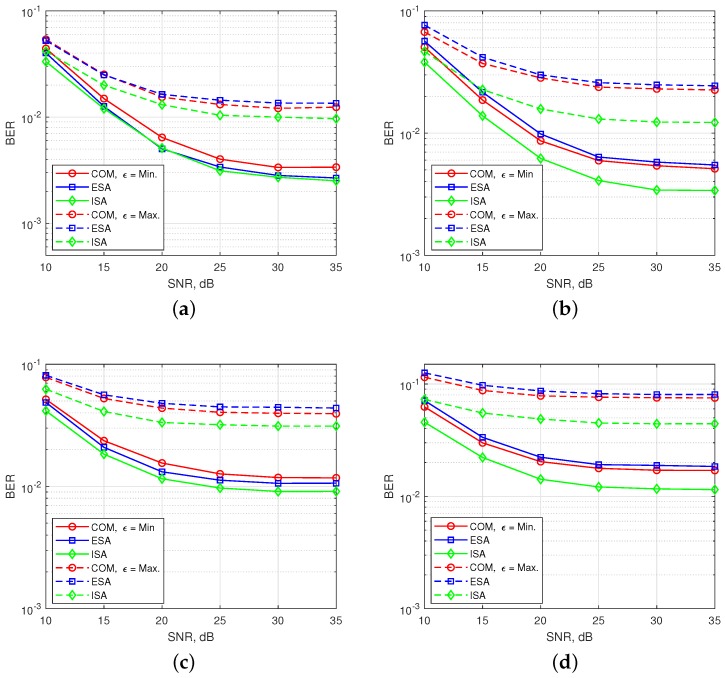
BER performance of multi-user OFDM-IM regarding to three SMS-s. Only time offset between the machine users is considered for (**a**) and (**b**), while both time and power offset are considered for (**c**) and (**d**). (**a**) SMS-s with s=8 and v=3. (**b**) SMS-s with s=8 and v=4. (**c**) SMS-s with s=8 and v=3. (**d**) SMS-s with s=8 and v=4.

In Figure 10, BER performances are obtained for 2-nd user under time and power misalignment. In Figure 10a,b, only time misalignment between the users is considered. As seen in the Figure 10a, ISA with (s=8, v=3) has the best BER performance, but with a slight difference from ESA for ϵ=Min. Edge subcarrier activation probability for ESA is higher in comparison to COM and ISA, as shown in Figure 6. Therefore, ICI coming from adjacent users to the 2-nd user further increases for ESA. For ϵ=Max, the difference between the performances of SMS-s is more obvious and ESA has the worst performance. COM has a slight better BER performance than ESA as observed in the Figure 8b. ISA offers a much better BER performance for maximum time misalignment, since it has the smallest edge subcarrier activation probability associated with the lowest ICI. In Figure 10b, OFDM-IM with subblock parameters (s=8, v=4) is simulated. The performance of both ESA and COM become much worse than in Figure 10a. For ISA with (s=8, v=4), subcarrier usage probability is more localized around the middle subcarriers than in the case of ISA with (s=8, v=3), as shown in Figure 6. Moreover, equiprobable activation properties of ESA causes a destructive effect on the BER performance due to non-uniform distribution of ICI. For COM, less activation probability of one edge provides better protection against ICI caused by asynchronism between the users in time. In Figure 10c,d, power difference between the users is also considered as well as time offset. The advantages of ISA against asynchronous transmission impairments are much more visible with the increase of ICI. Not only power difference but also increased number of active subcarriers within the OFDM-IM subblock results in higher ICI. Therefore, ISA mapping scheme plays a key role for larger subcarrier activation ratio of v/s in asynchronous mMTC networks.

## 6. Conclusions

One of the fundamental challenges for 5G and beyond technologies is to handle asynchronous impairments in uncoordinated mMTC networks. Performance of OFDM under time misalignment conditions is severely affected due to its susceptibility against ICI. Fractional subcarrier activation in OFDM-IM develops immunity to ICI caused by both time and power misalignments between the machine users. Flexible and adaptive structure of OFDM-IM provides opportunities to manage the active subcarriers. A novel subcarrier activation scheme ISA is proposed by considering the non-uniform distribution of ICI in asynchronous transmission. It offers the best performance in comparison to existing methods both COM and ESA without increasing the computational complexity. In addition, energy-free transmission through active subcarrier indices makes OFDM-IM technology a strong candidate for mMTC networks that require low energy consumption.

In this study, investigations are performed for OFDM-IM systems by considering asynchronous impact of mMTC networks. In order not to exceed the scope of this paper, OFDM-IM systems performance regarding other impacts of mMTC architecture is left for future studies. In the future work, we will consider both clustering users and conflicted users for mMTC. Moreover, optimization of OFDM-IM subblock size and activation ratio will be evaluated for different machine users with respect to their requirements.

## Figures and Tables

**Table 1:** Symbol description used in the paper.

Symbol	Description
$x_{u}\left(n\right)$	Transmitted signal by *u*-th user
$h_{u}\left(n\right)$	Channel belongs to *u*-th user
$\epsilon_{u}$	Time offset for *u*-th user
$y_{u}\left(n\right)$	Received Signal by *u*-th user
*N*	OFDM-IM block size
*G*	Number of OFDM-IM subbblock
*U*	Number of machine users
$N_{u}$	Number subcarriers per user
$G_{u}$	Number of subblocks per user
*s*	OFDM-IM subblock size
*v*	Number of active subcarriers within a OFDM-IM subbblock
$\xi_{u}^{l}$	Indices of active subcarriers of *u*-th user for *l*-th subblock
*p*	Number of bits per OFDM-IM subblock
p1	Bit stream corresponds to active subcarriers for a OFDM-IM subblock
p2	Bit stream is mapped to *M*-ary symbols
$m_{u}$	Number of transmitter per user
$c_{u}^{i}$	*i*-th data block of *u*-th user
${ti}_{u_{y}}^{u_{x}}$	Interference coming from $u_{x}$ to the $u_{y}$
$\Delta p_{u_{x}u_{y}}$	Power difference between $u_{x}$ and $u_{y}$
$I_{u}$	ICI for *u*-th user

**Table 2:** Look-up table for (s=4,v=2).

β	ξ	*I*
00	1,2	$\left[X_{l}^{1}\;X_{l}^{2}\;0\;0\right]$
01	2,3	$\left[0\;X_{2}^{1}\;X_{l}^{3}\;0\right]$
10	3,4	$\left[0\;0\;X_{l}^{3}\;X_{l}^{4}\right]$
11	1,4	$\left[X_{l}^{1}\;0\;0\;X_{l}^{4}\right]$

## Abbreviations

The following abbreviations are used in this study:

AWGN	Additive White Gaussian Noise
BS	Base Station
CFO	Carrier Frequecny Offset
COM	Combinatorial
CP	Cyclic Prefix
ESA	Equiprobable Subcarrier Activation
FBMC	Filter Bank Multi Carrier
FFT	Fast Fourier Transform
GFDM	Generalized Frequency Division Multiplexing
ICI	Inter-Carrier Interference
IFFT	Inverse-Fast Fourier Transform
IM	Index Modulation
ISA	Inner Subcarrier Activation
ISI	Inter Symbol Interference
LLR	Log-Likelihood Ratio
LUT	Look Up Table
MIMO	Multiple Input Multiple Output
ML	Maximum Likelihood
mMTC	Massive Machine-Type Communications
MUI	Multi User Interference
OFDM	Orthogonal Frequency Division Multiplexing
OFDM-IM	Orthogonal Frequency Division Multiplexing with Index Modulation
OOBE	Out-of-Band Emission
SA	Subblock Assignment
SNR	Signal to Noise Ratio
SM	Spatial Modulation
SMS	Subcarrier Mapping Scheme
TO	Time Offset
UFMC	Universal Filtered Multi-Carrier
V2X	Vehicle to X
